# New Highly Active Antiretroviral drugs and generic drugs for the treatment of HIV infection: a budget impact analysis on the Italian National Health Service (Lombardy Region, Northern Italy)

**DOI:** 10.1186/s12879-015-1077-7

**Published:** 2015-08-11

**Authors:** Umberto Restelli, Francesca Scolari, Paolo Bonfanti, Davide Croce, Giuliano Rizzardini

**Affiliations:** Centre for Research on Organisation, Innovation and Leadership in Healthcare (CROILS), IRCCS Ospedale San Raffaele, Milan, Italy; School of Public Health, Faculty of Health Sciences, University of the Witwatersrand, Johannesburg, South Africa; Department of Infectious and Tropical Diseases, A. Manzoni Hospital, Lecco, Italy; First and Second Divisions of Infectious Diseases, “Luigi Sacco” Hospital, Milan, Italy; School of Clinical Medicine, Faculty of Health Sciences, University of the Witwatersrand, Johannesburg, South Africa

## Abstract

**Background:**

In the healthcare sector, it is crucial to identify sustainable strategies in order to allow the introduction and use of innovative technologies. Now, and over the next few years, the expiry of patents for different antiretroviral drugs offers an opportunity to increase the efficiency of resources allocation. The aim of the present study was to assess the impact, on the budget of the Italian National Healthcare Service, of generic antiretroviral drugs and of new antiretroviral drugs entering the market from 2015 to 2019.

**Methods:**

A budget impact model was developed in order to forecast the rate of use of ARTs, based on trends observed within the Lombardy Region (Italy), on clinical experts’ opinion, and the consequent impact on the Italian NHS budget in a five year time horizon. Different scenarios were developed, considering the sole introduction of generic drugs, of new drugs, and their cumulative effects. A multivariate sensitivity analysis was also performed.

**Results:**

The cumulative use of generic drugs and new drugs would lead to annual savings of 4.6 million € (-0.6 %) in 2015; 16.9 million € (-2.1 %) in 2016; 19.4 million € (-2.4 %) in 2017; 51.1 million € (-6.1 %) in 2018 and -110.3 million € (-12.8 %) in 2019. The impact of new drugs in percentage terms is +2.0 % in 2015, +3.4 % in 2016, +3.9 % in 2017, +5.7 % in 2018 and +7.7 % in 2019. The impact of generic drugs would lead to savings of 4.9 million € in 2015, 18.6 million € in 2016, 22.8 million € in 2017, 76.5 million € in 2018 and 187.4 million € in 2019.

The sensitivity analysis showed annual mean savings for the Italian NHS ranging from 12.6 million €, -1.5 % compared to the base case scenario (decreasing all the rates of transition used in the simulation, and increasing the cost of generic drugs) to 76.0 million €, -9.1 % (increasing all the rates of transition used in the simulation, and decreasing the cost of generic and new drugs).

**Conclusions:**

The use of antiretroviral generic drugs may lead to savings that would compensate the expenditure increase due to new, innovative drugs available on the market.

**Electronic supplementary material:**

The online version of this article (doi:10.1186/s12879-015-1077-7) contains supplementary material, which is available to authorized users.

## Background

Since 2005, the availability of new antiretroviral drugs which offer high efficacy and acceptability and less toxicity, has led to a decrease of HIV related deaths worldwide [1, 2], compared with the pre Highly Active Antiretroviral Therapy (HAART) era. As a result of advances in treatment, HIV infection is now considered as a chronic disease [3]: patients have to follow Antiretroviral Therapies (ARTs) for several years, thus increasing the relevance of compliance, and exposing them to long-term adverse events.

Although progress has been substantial, uncertainties persist concerning the best way to manage HIV disease [4].

Health-care decision making and management of medical technologies and drugs is crucial in the healthcare sector, since innovative technologies often lead to an increase of costs [5–7]. This is the case with antiretroviral drugs, which are often more effective and more expensive (although in some cases the increased cost of drugs is compensated by a reduction of adverse events and, therefore of inpatient and outpatient activities for healthcare services). Sullivan and colleagues [8] showed how HIV, in terms of cost per person, was the third most expensive disease in France in 2005, with a cost per person of 11,900 €, above the 7,000 € mean expense for all diseases. In Italy, Magoni and colleagues [9] analysed the per capita cost in 2007 for the Healthcare Service of Lombardy Region of patients referring to a Local Health Authority with more than 1.1 million inhabitants. HIV infection was found to be the third most expensive chronic disease, considering direct medical costs (inpatient, outpatient activities and drugs) and the most expensive considering drugs alone.

Two further Italian studies conducted within the 1st and 2nd Infectious Diseases wards at Luigi Sacco Hospital, based in Milan, among 653 and 483 patients respectively, showed a constant yearly increase in the mean HAART costs in periods 2004 − 2007 and 2007 − 2009 [10, 11].

In such a scenario, the current economic recession has led some European and North American governments to adopt austerity policies, during a period in which countercyclical investments would be needed [12, 13] and concerns over the sustainability of health care expenditures have been raised.

To face this challenging situation an increase in the efficiency of resources allocation is needed, identifying cost saving strategies that ensure the efficacy of treatments.

Different approaches have been used among European countries, shifting expensive drugs to second line treatments, as within the French guidelines for antiretroviral treatments [14], shifting patients to less drugs regimen, encouraging cost-effectiveness criteria and promoting the use of generic drugs. Considering this strategy, over the next few years different patents for antiretroviral drugs will expire, thus allowing generic drugs to enter the market, with the positive effects of lowering the cost of treatments.

The aim of the present study was to assess the impact on budget of the Italian National Healthcare Service (INHS) of new generic antiretroviral drugs and of new antiretroviral drugs entering the market between 2015 and 2019. The budget impact of these two categories of antiretroviral treatments was investigated (both separately and in a cumulative way), in order to inquire if the savings due to the use of generic drugs could balance the increased cost of antiretroviral treatment resulting from the use of the new antiretroviral drugs.

## Methods

In order to assess the potential impact of generic drugs and new antiretroviral drugs on the budget of the INHS, a model was developed to forecast the rate of use of ARTs.

The trends in the rate of consumption of HIV antiretroviral drugs observed by the Lombardy Region HIV/AIDS group of technical experts between 2010 and 2012 were considered to forecast the use of ARTs in a five year time horizon, [15] from 2015 to 2019, based on clinical experts opinion. Input from experts were based on the direct clinical experience of clinicians referring to the Infectious Disease Department of two Hospital Authorities within the previously mentioned region.

The model structure considers cycles of six months. In order to forecast the use of each ART, the rate of consumption estimated in each semester was multiplied by the number of HIV infected subjects on ART, calculated considering the data provided by the Italian National Institute of Health [16].

A base case scenario was created, estimating the trends of consumption of the brand and generic antiretroviral drugs already being used in 2015. A comparative scenario was developed estimating the consumption of ART following the introduction of new brand drugs and of generic drugs: DTG, ABC/3TC/DTG, TAF/FTC/EVG/COBI, TAF/FTC/RPV, TAF/FTC, generic ABC, generic LPV, generic NVP, generic TDF/FTC/EFV, generic DRV, generic ATV. The estimated effects of each new drug on the rate of consumption of other drugs are reported in Table 1.

**Table 1:** The model’s base case hypotheses – effects of generic and new drugs

Drug	Semester in which it enters in the model	Effects [sensitivity analysis rates]
Generic ABC	First semester 2015	100 % of patients receiving branded ABC switch to generic ABC in the semester in which the new drug enter the model [85 %; 100 %]
		10 % of patients receiving ABC/3TC switch to generic 3TC + generic ABC in the semester in which the new drug enter the model [5 %; 25 %]
Generic LPV	First semester 2016	100 % of patients receiving branded LPV switch to generic LPV in the semester in which the new drug enter the model [85 %; 100 %]
Generic NVP 400	Second semester 2016	100 % of patients receiving branded NVP switch to generic NVP in the semester in which the new drug enter the model [85 %; 100 %]
Generic TDF/FTC/EFV	Second semester 2018	60 % of patients receiving branded TDF/FTC/EFV switch to generic TDF/FTC/EFV [50 %; 100 %]
		35 % of patients receiving branded TDF/FTC/EFV should switch to generic TDF/FTC/EFV, however clinicians prefer a switch to TAF/FTC/RPV following indications of clinical pathway [25 %; 0 %]
		5 % of patients receiving branded TDF/FTC/EFV should switch to generic TDF/FTC/EFV, however clinicians prefer a switch to TAF/FTC/EVG/COBI following indications of clinical pathway [0 %; 0 %]
Generic DRV	Second semester 2018	50 % of patients receiving branded DRV + TDF/FTC switch to generic DRV + TAF/FTC [40 %; 100 %]
		15 % of patients receiving branded DRV + TDF/FTC should switch to generic DRV + TAF/FTC, however clinicians prefer a switch to TAF/FTC/EVG/COBI following indications of clinical pathway [5 %; 0 %]
		15 % of patients receiving branded DRV + TDF/FTC should switch to generic DRV + TAF/FTC, however clinicians prefer a switch to TAF/FTC + DTG following indications of clinical pathway [5 %; 0 %]
		20 % of patients receiving branded DRV + TDF/FTC should switch to generic DRV + TAF/FTC, however clinicians prefer a switch to TAF/FTC/RPV following indications of clinical pathway [10 %; 0 %]
		DRV + ABC/3TC switch to generic DRV + ABC/3TC [40 %; 100 %]
		50 % of patients receiving branded DRV + ABC/3TC should switch to generic DRV + ABC/3TC, however clinicians prefer a switch to ABC/3TC/DTG following indications of clinical pathway [40 %; 0 %]
Generic ATV	First semester 2019	50 % of patients receiving branded ATV + TAF/FTC switch to generic ATV + TAF/FTC[40 %; 100 %]
		15 % of patients receiving branded ATV + TDF/FTC should switch to generic ATV + TAF/FTC, however clinicians prefer a switch to TAF/FTC/EVG/COBI following indications of clinical pathway [5 %; 0 %]
		15 % of patients receiving branded ATV + TDF/FTC should switch to generic ATV + TAF/FTC, however clinicians prefer a switch to TAF/FTC + DTG following indications of clinical pathway [5 %; 0 %]
		20 % of patients receiving branded ATV + TDF/FTC should switch to generic ATV + TAF/FTC, however clinicians prefer a switch to TAF/FTC/RPV following indications of clinical pathway [10 %; 0 %]
		50 % of patients receiving branded ATV + ABC/3TC switch to generic ATV + ABC/3TC [40 %; 100 %]
		50 % of patients receiving branded ATV + ABC/3TC should switch to generic ATV + ABC/3TC, however clinicians prefer a switch to ABC/3TC/DTG following indications of clinical pathway [40 %; 0 %]
DTG	First semester 2015	10 % of switching patients receiving DRV (0.78 %) switch to DTG in each semester [5 %; 25 %]
		10 % of switching patients receiving ATV (0.78 %) switch to DTG in each semester [5 %; 25 %]
		75 % of switching patients receiving RAL (5.87 %) switch to DTG in each semester [50 %; 75 %]
ABC/3TC/DTG	Second semester 2015	100 % of patients receiving ABC/3TC + DTG switch to ABC/3TC/DTG in the semester in which the new drug enter the model [85 %; 100 %]
		20 % of patients receiving TDF/FTC + DTG switch to ABC/3TC/DTG in the semester in which the new drug enter the model [10 %; 30 %]
TAF/FTC/EVG/COBI	Second semester 2016	100 % of patients receiving TDF/FTC/EVG/COBI switch to TAF/FTC/EVG/COBI in the semester in which the new drug enter the model [100 %; 100 %]
TAF/FTC	First semester 2017	100 % of patients receiving TDF/FTC switch to TAF/FTC in the semester in which the new drug enter the model [100 %; 100 %]
TAF/FTC/RPV	Second semester 2017	100 % of patients receiving TDF/FTC/RPV switch to TAF/FTC/RPV in the semester in which the new drug enter the model [100 %; 100 %]

Abbreviations: *3TC* Lamivudine, *EFV* Efavirenz, *TDF* Tenofovir Disoproxil Fumarate, *FTC* Emtricitabine, *NVP* Nevirapine, *ABC* Abacavir, *LPV* Lopinavir, *DTG* Dolutegravir, *DRV* Darunavir, *RAL* Raltegravir, *EVG* Elvitegravir, *COBI* Cobicistat, *RPV* Rilpivirine, *TAF* Tenofovir Alafenamide Fumarate. Table legend text

Regional clinical pathways in Italy suggest the use of single tablet regimens, where possible. Therefore, in the definition of the therapy switch strategies, the availability of multiple generic pills had a limited effect on the rate of use of co-formulated tablet, to preserve the compliance of the patients.

The analysis was also performed considering the impact of the two categories of new therapies (new drugs and generic drugs) alone. The semester in which each generic drug entered the model was the same as that of the expiration date of the drug complementary protection certificate. In year 1 (2015) patients were allocated within the therapies considered in the model, as estimated through the rate of drugs consumption observed. The “other therapies” category included all the patients not receiving any of the therapies considered in the model (Additional file 1).

The cost considered was that of the antiretroviral drugs, referred to 2015 levels, as reported in the HIV/AIDS Clinical Pathway of the Lombardy Region [17] and was undiscounted [15] (presented in Table 2). The cost per patient per semester was calculated by multiplying the monthly therapy cost by 5.25, due to the fact that the reprocessing of the data within the Lombardy Region HIV/AIDS group of technical experts showed (due to a lack of complete compliance) a per patient yearly mean consumption of HIV ART daily doses of 10.5 months.

**Table 2:** Drugs prices considered in the analysis as reported within the HIV/AIDS Clinical Pathway of the Lombardy Region

Drug	Daily dose	Brand drug monthly cost (€)	Generic drug monthly cost (€)	Source for brand price
ABC 300 mg	2	224.40	89.76	[17]
ATV 200 mg	2	503.58	201.43	[17]
ATV 300 mg	1	332.97	133.19	[17]
DRV 400 mg	2	348.48	139.39	[17]
DRV 600 mg	2	528.00	211.20	[17]
DTG 50 mg	1	495.15	-	[17]
EFV 600 mg	1	128.68	128.68	[17]
FTC 200 mg	1	161.37	64.55	[17]
3TC 300 mg or 150 mg	1 (300 mg) or 2 (150 mg)	25.74	25.74	[17]
NVP 400 mg	1	178.53	71.41	[17]
RAL 400 mg	2	438.90	-	[17]
RPV 25 mg	1	230.67	-	[17]
Ritonavir 100 mg	1 or 2	24.97 (100 mg)	-	[17]
TDF 245 mg	1	276.87	-	[17]
TAF 245 mg	1	276.87	-	Expert opinion
TDF/FTC 200/245 mg	1	438.90	-	[17]
TAF/FTC 200/245 mg	1	438.90	-	Expert opinion
ABC/3TC 600/300 mg	1	398.31	-	[17]
3TC/AZT 150/300 mg	2	66.00	66.00	[17]
LPV/r 200/50 mg	4	357.72	143.09	[17]
ABC/3TC/AZT 300/150/300 mg	2	500.28	-	[17]
ABC/3TC/DTG 600/300/50 mg	1	893.46	-	Expert opinion
FTC/TDF/EFV 200/245/600 mg	1	596.70	238.68	[17]
FTC/TDF/RPV 200/245/25 mg	1	598.62	-	[17]
FTC/TAF/RPV 200/245/25 mg	1	598.62	-	Expert opinion
EVG/COBI/FTC/TDF 150/150/200/245 mg	1	797.61	-	[17]
EVG/COBI/FTC/TAF 150/150/200/245 mg	1	797.61	-	Expert opinion

The generic drugs cost was estimated to be 40 % of the branded cost. The three generic antiretroviral drugs available in 2015 in Italy, lead in Lombardy Region to a mean reduction compared to the branded drugs price of 63.0 %, as observed within HIV/AIDS clinical pathways [17–19], therefore we approximate to 60 % the cost reduction. The cost of new drugs were considered at the same level of their main competitor or adding the cost of each active ingredient. The cost per patient of “other therapies” was calculated by subtracting the cost of the ART considered in the model from the INHS ART expenditures [20]. The result was divided by the number of patients within this category.

A sensitivity analysis was also conducted in order to assess the robustness of the results. The parameters changed were the price of generic drugs (20 % of brand drugs price; and 60 % of brand drug price), the price of new drugs (-10 % than the base case scenario price) and the rate of use of generic drugs and of new drugs, as reported in Table 1.

An increase in the number of HIV positive patients treated with ART was estimated in the analysis (based on data on new diagnosis provided by the Italian National Health Institute) [21], being 94,727 in the second semester of 2015, 99,205 in the second semester of 2016, 103,893 in the second semester of 2017, 108,804 in the second semester of 2018 and 113,946 in the second semester of 2019.

## Results

The consumption of generic drugs increased each semester, starting from a number of patients receiving at least one generic drug equal to 12,846 in the second semester of 2015, to 16,035 in the second semester of 2016, to 16,851 in the second semester of 2017, to 34,946 in the second semester of 2018 and to 50,866 in the second semester of 2019. In the last semester of the analysis, the number of patients receiving at least one generic drug represents 44.6 % of the number of HIV positive patients receiving ART.

The yearly cost of ART to treat the whole population of HIV positive patients for the INHS in the two scenarios (without new and generic drugs, and with new and generic drugs), the cost per capita and the yearly percentage differences are reported in Table 3.

**Table 3:** The model’s results considering the cumulative effect of new and generic drugs

		2015	2016	2017	2018	2019
Number of patients		94,727	99,205	103,893	108,804	113,946
No new and generic drugs scenario	Total ART cost (€)	764,962,036	789,011,152	813,397,033	838,115,496	863,159,214
	Per capita ART cost (€)	8,075	7,953	7,829	7,703	7,575
New and generic drugs scenario	Total ART cost (€)	760,408,704	772,152,627	794,021,079	787,013,681	752,848,759
	Per capita ART cost (€)	8,027	7,783	7,643	7,233	6,607
Δ in total cost (€)		−4,553,331	−16,858,525	−19,375,954	−51,101,815	−110,310,454
% Δ in total cost		−0.6 %	−2.1 %	−2.4 %	−6.1 %	−12.78 %

The percentage savings for the INHS are -0.6 % in 2015, -2.1 % in 2016, -2.4 % in 2017, -6.1 % in 2018 and -12.8 % in 2019, corresponding respectively to 4.6 million €, 16.9 million €, 19.4 million €, 51.1 million € and 110.3 million €.

Considering the per capita yearly ART cost, the scenario that does not consider new and generic drugs shows a linear cost decrease from 8,0975 € in year 2015 to 7.575 € in 2019. The per capita ART cost in the scenario that considers new and generic drugs led to a yearly cost decrease (compared with the previous year) of, -3.0 % in 2016, -1.8 % in 2017, -5.4 % 2018 and -8.7 in 2019.

Further analyses were performed, isolating the effects of new antiretroviral drugs and of generic drugs on the INHS budget. Considering the previously mentioned categories alone, the differential costs, compared with the base case scenario are reported in Table 4.

**Table 4:** The model’s results considering the effect of new and generic drugs alone

		2015	2016	2017	2018	2019
Number of patients		94,727	99,205	103,893	108,804	113,946
New antiretroviral drugs scenario	Total ART cost (€)	780,148,483	815,891,938	845,063,294	885,581,470	929,655,763
	Per capita ART cost (€)	8,236	8,224	8,134	8,139	8,159
	Δ in total cost (€) compared to base case	+15,186,448	+26,880,786	+31,666,260	+47,465,974	+66,496,550
	% Δ in total cost	+2.0 %	+3.4 %	+3.9 %	+5.7 %	+7.7 %
Generic drugs scenario	Total ART cost (€)	759,981,491	770,436,043	790,580,416	761,583,789	675,775,852
	Per capita ART cost (€)	8,023	7,766	7,610	7,000	5,931
	Δ in total cost (€)	−4,980,545	−18,575,109	−22,816,617	−76,531,708	−187,383,361
	% Δ in total cost	−0.7 %	−2.4 %	−2.8 %	−9.1 %	−21.7 %

**Table 5:** Sensitivity analysis results: percentage difference between “New and generic drugs scenarios”, and the scenario that does not consider new and generic drugs

Scenario	2015	2016	2017	2018	2019	5 Years mean difference
Base case	−0.6 %	−2.1 %	−2.4 %	−6.1 %	−12.8 %	−4.8 %
Increased costs	−0.5 %	−1.4 %	−1.3 %	−3.9 %	−8.4 %	−3.1 %
Decreased costs	−0.7 %	−3.0 %	−3.6 %	−9.1 %	−19.0 %	−7.1 %
Increased switch rates	−2.3 %	−3.9 %	−4.2 %	−7.7 %	−14.6 %	−6.6 %
Decreased switch rates	0.2 %	−0.7 %	−0.9 %	−4.0 %	−9.6 %	−3.0 %
Increased costs and decreased switch rates	0.2 %	−0.1 %	0.0 %	−1.9 %	−5.5 %	−1.5 %
Increased costs and switch rates	−2.1 %	−3.0 %	−3.0 %	−5.3 %	−9.7 %	−4.6 %
Decreased costs and switch rates	0.2 %	−1.2 %	−1.6 %	−6.2 %	−14.4 %	−4.7 %
Decreased costs and increased switch rates	−2.9 %	−5.3 %	−5.9 %	−10.9 %	−20.7 %	−9.1 %

The impact of new drugs in percentage terms was +2.0 % in 2015, +3.4 % in 2016, +3.9 in 2017, +5.7 % in 2018 and +7.7 % in 2019.

Different results were obtained considering generic drugs only. The impact of these technologies on the INHS budget would lead to savings in years 2015 − 2019 of -5.0 million € in 2015, -18.6 million € in 2016, -22.8 million € in 2017, 76.5 million € in 2018 and -187.4 million € in 2019.

**Fig. 1:**
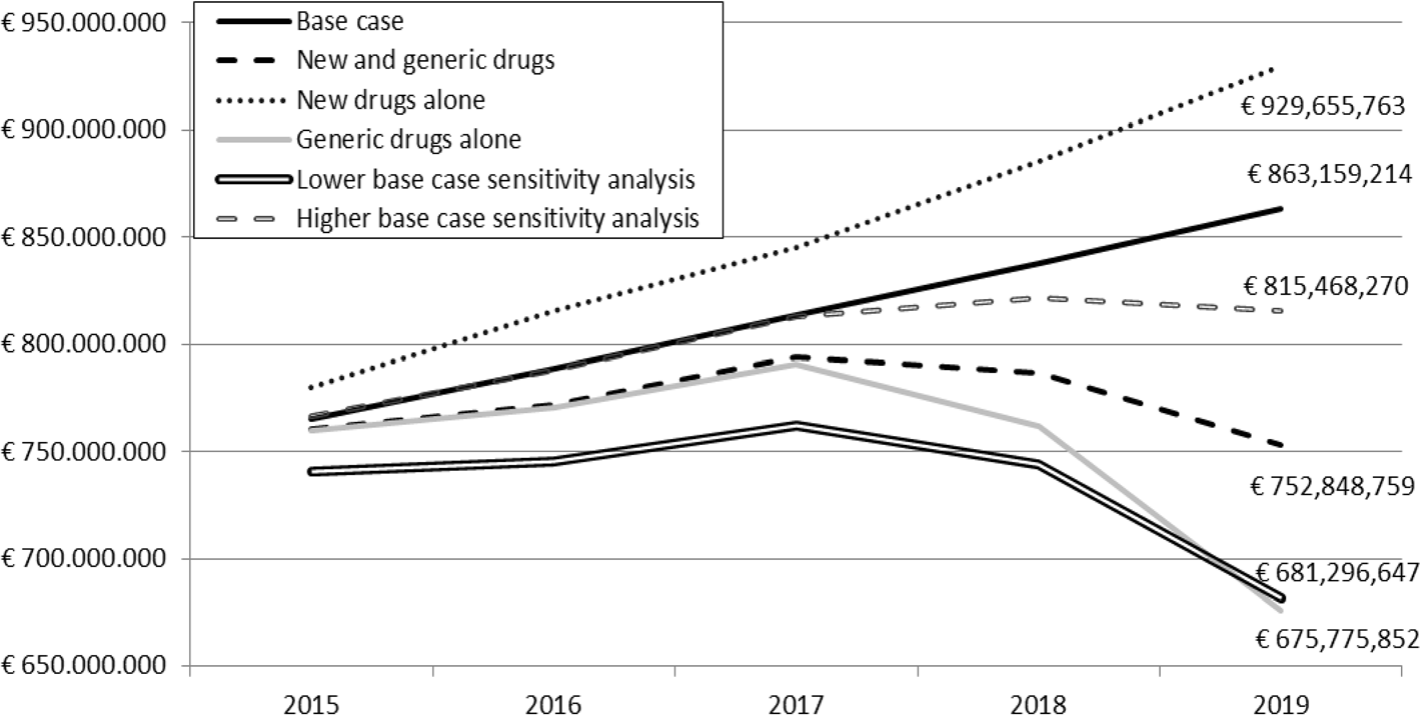
Total cost of the 4 scenarios and sensitivity analysis range of the “new and generic antiretroviral drugs” scenario

The yearly total cost of each scenario is presented in Fig. 1.

The sensitivity analysis results, showed annual mean savings for the INHS ranging from 12.6 million €, -1.5 % compared to the base case scenario (decreasing all the rates of transition used in the simulation, and increasing the cost of generic drugs) to 76.0 million €, -9.1 % (increasing all the rates of transition used in the simulation, and decreasing the cost of generic and new drugs) (Table 5).

## Discussion

The actual context of resources constraints has forced national health services to design strategies in order to increase the efficiency of resources allocation and treatment appropriateness. Among the possible strategies to contain costs for the management of HIV positive patients, without affecting the efficacy of therapies, the use of generic drugs is one of the most feasible, since it does not affect the patient pathway in terms of increased outpatients activities. Due to the increase in patient’s survival years, a new paradigm for the management of HIV positive patients has been established. The use of effective therapies with high genetic barrier increase the future therapeutic options for patients and are, therefore, to be considered as first line therapies. However, new drugs with the aforementioned characteristics have high costs, compared to the therapies already available on the market.

The present study shows how the savings due to cost containing strategies (such as the use of generic drugs) would allow investment in more expensive drugs, with possible future savings related to the increased range of therapies to be administered once the first ART leads to a virological failure.

The results show savings related to the use of generic drugs, in the same range of previously published data related to the Italian context [22] (for Lazio Region), while data published concerning the U.K. [23], estimate further savings, around 20 % in years 2014, 2015 and 2016, and around 70 % in years 2017 and 2018 (even though the price reduction of generic drugs, in the study presented by Hill and colleagues, is higher than the hypothesis made in the present study, and the years considered in the two studies are different).

Finally, although the findings suggest that the use of generic drugs could generate significant cost savings, there were limitations with the present study. The analysis performed focused on ART costs and did not consider hospitalizations, outpatient activities and other drugs, and the likely fewer activities due to the use of new drugs, leading to further savings.

The conservative assumptions of the model were also related to the fact that the analysis was based on a comparative scenario taken from the Lombardy Region, that has been able to control, over the past years, the per capita costs of HIV positive patients through the implementation of a clinical pathway. Therefore, comparing the results of the analysis with such an efficient scenario, may have underestimated the advantages at a National level.

## Conclusions

In conclusion, the use of generic antiretroviral drugs within the INHS may lead to savings, compensating for any expenditure increase due to the use of new, innovative drugs available on the market. The identification of cost containing strategies, such as the one presented in the present study, would thus allow the sustainability of the use of new drugs at a time of increased budget constraints.

## Additional file

Additional file 1:
**Patient’s distribution in the second semester of each year of the base case scenario and of the “new and generic drugs” scenario.** (DOCX 39 kb)
