# Supplementary material for: New Highly Active Antiretroviral drugs and generic drugs for the treatment of HIV infection: a budget impact analysis on the Italian National Health Service (Lombardy Region, Northern Italy)
Source: BMC Infect Dis. 2015 Aug 11;15:323. doi: 10.1186/s12879-015-1077-7 (PMC4531431; doi:10.1186/s12879-015-1077-7)
Supplement: Additional file 1: — Patient’s distribution in the second semester of each year of the base case scenario and of the “new and generic drugs” scenario. (DOCX 39 kb) [file 12879_2015_1077_MOESM1_ESM.docx]

Supplementary material

Patient’s distribution in the second semester of each year of the base case scenario and of the “new and generic drugs” scenario

| **Therapies** | **Base case scenario** | | | | | | **New and generic drugs scenario** | | | | |
| --- | --- | --- | --- | --- | --- | --- | --- | --- | --- | --- | --- |
|  | **2014** | **2015** | **2016** | **2017** | **2018** | **2019** | **2015** | **2016** | **2017** | **2018** | **2019** |
| TDF/FTC/EFV | 15,0% | 16,0% | 17,0% | 18,0% | 19,0% | 20,0% | 15,7% | 16,4% | 17,1% | 0,0% | 0,0% |
| TDF/FTC/EFV (generic) | 0,0% | 0,0% | 0,0% | 0,0% | 0,0% | 0,0% | 0,0% | 0,0% | 0,0% | 11,0% | 12,0% |
| TDF/FTC + EFV (generic) | 5,5% | 5,7% | 5,9% | 6,1% | 6,3% | 6,5% | 5,7% | 5,9% | 0,0% | 0,0% | 0,0% |
| TAF/FTC + EFV (generic) | 0,0% | 0,0% | 0,0% | 0,0% | 0,0% | 0,0% | 0,0% | 0,0% | 6,1% | 6,3% | 6,5% |
| 3TC/ABC + EFV (generic) | 1,4% | 1,6% | 1,8% | 2,0% | 2,2% | 2,4% | 1,4% | 1,6% | 1,8% | 2,0% | 2,2% |
| 3TC (generic) + ABC (generic) + EFV (generic) | 0,0% | 0,0% | 0,0% | 0,0% | 0,0% | 0,0% | 0,1% | 0,1% | 0,1% | 0,3% | 0,5% |
| 3TC/AZT (generic) + EFV (generic) | 0,9% | 0,9% | 0,9% | 0,9% | 0,9% | 0,9% | 0,9% | 0,9% | 0,9% | 0,9% | 0,9% |
| TDF/FTC + LPV | 1,0% | 0,9% | 0,8% | 0,7% | 0,6% | 0,5% | 0,9% | 0,0% | 0,0% | 0,0% | 0,0% |
| TAF/FTC + LPV | 0,0% | 0,0% | 0,0% | 0,0% | 0,0% | 0,0% | 0,0% | 0,0% | 0,0% | 0,0% | 0,0% |
| TDF/FTC + LPV (generic) | 0,0% | 0,0% | 0,0% | 0,0% | 0,0% | 0,0% | 0,0% | 0,8% | 0,0% | 0,0% | 0,0% |
| TAF/FTC + LPV (generic) | 0,0% | 0,0% | 0,0% | 0,0% | 0,0% | 0,0% | 0,0% | 0,0% | 0,7% | 0,6% | 0,5% |
| 3TC/ABC (brand) + LPV | 0,9% | 0,8% | 0,7% | 0,6% | 0,5% | 0,4% | 0,7% | 0,0% | 0,0% | 0,0% | 0,0% |
| 3TC/ABC (brand) + LPV (generic) | 0,0% | 0,0% | 0,0% | 0,0% | 0,0% | 0,0% | 0,0% | 0,6% | 0,5% | 0,4% | 0,4% |
| 3TC (generic) + ABC (generic) + LPV | 0,0% | 0,0% | 0,0% | 0,0% | 0,0% | 0,0% | 0,1% | 0,0% | 0,0% | 0,0% | 0,0% |
| 3TC (generic) + ABC (generic) + LPV (generic) | 0,0% | 0,0% | 0,0% | 0,0% | 0,0% | 0,0% | 0,0% | 0,1% | 0,1% | 0,1% | 0,1% |
| 3TC/AZT (generic) + LPV | 1,4% | 1,4% | 1,4% | 1,4% | 1,4% | 1,4% | 1,4% | 0,0% | 0,0% | 0,0% | 0,0% |
| 3TC/AZT (generic) + LPV (generic) | 0,0% | 0,0% | 0,0% | 0,0% | 0,0% | 0,0% | 0,0% | 1,3% | 1,3% | 1,3% | 1,2% |
| TDF/FTC + ATV | 14,5% | 14,2% | 13,9% | 13,6% | 13,3% | 13,1% | 14,0% | 13,5% | 0,0% | 0,0% | 0,0% |
| TAF/FTC + ATV | 0,0% | 0,0% | 0,0% | 0,0% | 0,0% | 0,0% | 0,0% | 0,0% | 13,0% | 12,5% | 0,0% |
| TDF/FTC + ATV (generic) | 0,0% | 0,0% | 0,0% | 0,0% | 0,0% | 0,0% | 0,0% | 0,0% | 0,0% | 0,0% | 0,0% |
| TAF/FTC + ATV (generic) | 0,0% | 0,0% | 0,0% | 0,0% | 0,0% | 0,0% | 0,0% | 0,0% | 0,0% | 0,0% | 6,2% |
| 3TC/ABC (brand) + ATV | 6,0% | 5,9% | 5,8% | 5,7% | 5,6% | 5,5% | 5,2% | 5,0% | 4,9% | 4,7% | 0,0% |
| 3TC/ABC (brand) + ATV (generic) | 0,0% | 0,0% | 0,0% | 0,0% | 0,0% | 0,0% | 0,0% | 0,0% | 0,0% | 0,0% | 2,3% |
| 3TC (generic) + ABC (generic) + ATV | 0,0% | 0,0% | 0,0% | 0,0% | 0,0% | 0,0% | 0,6% | 0,6% | 0,6% | 0,5% | 0,0% |
| 3TC (generic) + ABC (generic) + ATV (generic) | 0,0% | 0,0% | 0,0% | 0,0% | 0,0% | 0,0% | 0,0% | 0,0% | 0,0% | 0,0% | 0,3% |
| 3TC/AZT (generic) + ATV | 1,7% | 1,7% | 1,6% | 1,6% | 1,6% | 1,5% | 1,6% | 1,6% | 1,5% | 1,5% | 0,0% |
| 3TC/AZT (generic) + ATV (generic) | 0,0% | 0,0% | 0,0% | 0,0% | 0,0% | 0,0% | 0,0% | 0,0% | 0,0% | 0,0% | 1,4% |
| TDF/FTC + DRV 600 | 5,9% | 5,7% | 5,4% | 5,2% | 4,9% | 4,7% | 5,6% | 5,2% | 0,0% | 0,0% | 0,0% |
| TAF/FTC + DRV 600 | 0,0% | 0,0% | 0,0% | 0,0% | 0,0% | 0,0% | 0,0% | 0,0% | 4,9% | 0,0% | 0,0% |
| TDF/FTC + DRV 600 (generic) | 0,0% | 0,0% | 0,0% | 0,0% | 0,0% | 0,0% | 0,0% | 0,0% | 0,0% | 0,0% | 0,0% |
| TAF/FTC + DRV 600 (generic) | 0,0% | 0,0% | 0,0% | 0,0% | 0,0% | 0,0% | 0,0% | 0,0% | 0,0% | 2,4% | 2,1% |
| 3TC/ABC (brand) + DRV 600 | 1,4% | 1,4% | 1,3% | 1,3% | 1,2% | 1,2% | 1,2% | 1,2% | 1,1% | 0,0% | 0,0% |
| 3TC/ABC (brand) + DRV 600 (generic) | 0,0% | 0,0% | 0,0% | 0,0% | 0,0% | 0,0% | 0,0% | 0,0% | 0,0% | 0,5% | 0,5% |
| 3TC (generic) + ABC (generic) + DRV 600 | 0,0% | 0,0% | 0,0% | 0,0% | 0,0% | 0,0% | 0,1% | 0,1% | 0,1% | 0,0% | 0,0% |
| 3TC (generic) + ABC (generic) + DRV 600 (generic) | 0,0% | 0,0% | 0,0% | 0,0% | 0,0% | 0,0% | 0,0% | 0,0% | 0,0% | 0,1% | 0,1% |
| 3TC/AZT (generic) + DRV 600 | 0,2% | 0,2% | 0,2% | 0,2% | 0,2% | 0,2% | 0,2% | 0,2% | 0,2% | 0,0% | 0,0% |
| 3TC/AZT (generic) + DRV 600 (generic) | 0,0% | 0,0% | 0,0% | 0,0% | 0,0% | 0,0% | 0,0% | 0,0% | 0,0% | 0,2% | 0,2% |
| TDF/FTC + DRV 400 | 2,5% | 2,9% | 3,3% | 3,7% | 4,1% | 4,5% | 2,9% | 3,2% | 0,0% | 0,0% | 0,0% |
| TAF/FTC + DRV 400 | 0,0% | 0,0% | 0,0% | 0,0% | 0,0% | 0,0% | 0,0% | 0,0% | 3,6% | 0,0% | 0,0% |
| TAF/FTC + DRV 400 (generic) | 0,0% | 0,0% | 0,0% | 0,0% | 0,0% | 0,0% | 0,0% | 0,0% | 0,0% | 1,9% | 2,2% |
| 3TC/AZT (generic) + DRV 400 | 0,1% | 0,1% | 0,1% | 0,1% | 0,1% | 0,1% | 0,1% | 0,1% | 0,1% | 0,0% | 0,0% |
| 3TC/AZT (generic) + DRV 400 (generic) | 0,0% | 0,0% | 0,0% | 0,0% | 0,0% | 0,0% | 0,0% | 0,0% | 0,0% | 0,1% | 0,1% |
| TDF/FTC + NPV | 7,6% | 7,6% | 7,6% | 7,6% | 7,6% | 7,6% | 7,6% | 3,8% | 0,0% | 0,0% | 0,0% |
| 3TC/ABC (brand) + NPV | 4,9% | 4,9% | 4,9% | 4,9% | 4,9% | 4,9% | 4,9% | 2,5% | 0,0% | 0,0% | 0,0% |
| 3TC (generic) + ABC (generic) + NPV | 0,0% | 0,0% | 0,0% | 0,0% | 0,0% | 0,0% | 0,5% | 0,2% | 0,0% | 0,0% | 0,0% |
| TDF/FTC + NPV (generic) | 0,0% | 0,0% | 0,0% | 0,0% | 0,0% | 0,0% | 0,0% | 3,8% | 0,0% | 0,0% | 0,0% |
| TAF/FTC + NPV (generic) | 0,0% | 0,0% | 0,0% | 0,0% | 0,0% | 0,0% | 0,0% | 0,0% | 7,6% | 7,6% | 7,6% |
| 3TC/ABC (brand) + NPV (generic) | 0,0% | 0,0% | 0,0% | 0,0% | 0,0% | 0,0% | 0,0% | 2,5% | 4,9% | 4,9% | 4,9% |
| 3TC (generic) + ABC (generic) + NPV (generic) | 0,0% | 0,0% | 0,0% | 0,0% | 0,0% | 0,0% | 0,0% | 0,2% | 0,5% | 0,5% | 0,5% |
| 3TC/AZT (brand) + NVP (generic) | 0,0% | 0,0% | 0,0% | 0,0% | 0,0% | 0,0% | 0,0% | 0,0% | 0,0% | 0,0% | 0,0% |
| 3TC/AZT (generic) + NVP (generic) | 0,0% | 0,0% | 0,0% | 0,0% | 0,0% | 0,0% | 0,0% | 0,0% | 0,0% | 0,0% | 0,0% |
| TDF/FTC + RAL | 1,6% | 1,6% | 1,7% | 1,7% | 1,8% | 1,9% | 1,5% | 1,5% | 0,0% | 0,0% | 0,0% |
| TAF/FTC + RAL | 0,0% | 0,0% | 0,0% | 0,0% | 0,0% | 0,0% | 0,0% | 0,0% | 1,4% | 1,4% | 1,3% |
| 3TC/ABC (brand) + RAL | 1,2% | 1,3% | 1,4% | 1,4% | 1,5% | 1,5% | 1,1% | 1,1% | 1,1% | 1,0% | 1,0% |
| 3TC (generic) + ABC (generic) + RAL | 0,0% | 0,0% | 0,0% | 0,0% | 0,0% | 0,0% | 0,1% | 0,1% | 0,1% | 0,1% | 0,1% |
| 3TC/AZT (brand) + RAL | 0,0% | 0,1% | 0,2% | 0,2% | 0,3% | 0,3% | 0,1% | 0,1% | 0,2% | 0,2% | 0,3% |
| 3TC/AZT (generic) + RAL | 0,7% | 0,7% | 0,7% | 0,7% | 0,7% | 0,7% | 0,7% | 0,6% | 0,6% | 0,6% | 0,5% |
| DRV + RAL | 2,7% | 2,8% | 2,9% | 2,9% | 3,0% | 3,0% | 2,6% | 2,5% | 2,4% | 2,3% | 0,0% |
| DRV (generic) + RAL | 0,0% | 0,0% | 0,0% | 0,0% | 0,0% | 0,0% | 0,0% | 0,0% | 0,0% | 0,0% | 2,2% |
| ATV + RAL | 0,6% | 0,7% | 0,8% | 0,8% | 0,9% | 0,9% | 0,7% | 0,7% | 0,7% | 0,7% | 0,0% |
| ATV (generic) + RAL | 0,0% | 0,0% | 0,0% | 0,0% | 0,0% | 0,0% | 0,0% | 0,0% | 0,0% | 0,0% | 0,7% |
| LPV + RAL | 0,9% | 1,0% | 1,0% | 1,1% | 1,1% | 1,2% | 0,9% | 0,0% | 0,0% | 0,0% | 0,0% |
| LPV (generic) + RAL | 0,0% | 0,0% | 0,0% | 0,0% | 0,0% | 0,0% | 0,0% | 0,9% | 0,9% | 0,9% | 0,9% |
| ABC/3TC/AZT | 2,6% | 2,6% | 2,6% | 2,6% | 2,6% | 2,6% | 2,6% | 2,6% | 2,6% | 2,6% | 2,6% |
| TDF/FTC/RPV | 2,4% | 2,7% | 3,0% | 3,3% | 3,6% | 3,9% | 2,7% | 3,1% | 1,7% | 0,0% | 0,0% |
| TAF/FTC/RPV | 0,0% | 0,0% | 0,0% | 0,0% | 0,0% | 0,0% | 0,0% | 0,0% | 1,7% | 11,6% | 14,1% |
| TDF/FTC/EVG/COBI | 0,2% | 0,5% | 0,8% | 1,1% | 1,4% | 1,7% | 0,5% | 0,4% | 0,0% | 0,0% | 0,0% |
| TAF/FTC/EVG/COBI | 0,0% | 0,0% | 0,0% | 0,0% | 0,0% | 0,0% | 0,0% | 0,4% | 1,2% | 4,5% | 6,5% |
| TDF/FTC + DTG | 0,0% | 0,0% | 0,0% | 0,0% | 0,0% | 0,0% | 0,2% | 0,3% | 0,0% | 0,0% | 0,0% |
| TAF/FTC + DTG | 0,0% | 0,0% | 0,0% | 0,0% | 0,0% | 0,0% | 0,0% | 0,0% | 0,5% | 1,9% | 3,8% |
| 3TC/ABC (brand) + DTG | 0,0% | 0,0% | 0,0% | 0,0% | 0,0% | 0,0% | 0,0% | 0,0% | 0,0% | 0,0% | 0,1% |
| 3TC/AZT (generic) + DTG | 0,0% | 0,0% | 0,0% | 0,0% | 0,0% | 0,0% | 0,1% | 0,1% | 0,2% | 0,2% | 0,3% |
| 3TC/ABC/DTG | 0,0% | 0,0% | 0,0% | 0,0% | 0,0% | 0,0% | 0,3% | 0,5% | 0,7% | 1,0% | 3,7% |
| Other therapies | 16,3% | 14,5% | 12,8% | 10,9% | 9,1% | 7,1% | 14,9% | 13,7% | 12,8% | 11,0% | 9,3% |
